# Response to excessive selenium intake in the modern sow; a retrospective analysis of a case review

**DOI:** 10.1093/tas/txag048

**Published:** 2026-04-29

**Authors:** Sunniva Molnes, Hilde Vinje, Margareth Opheim, Erik Georg Granquist, Nicole Nyquist

**Affiliations:** Department of Paraclinical Sciences, Faculty of Veterinary Medicine, Norwegian University of Life Sciences, Ås, 1430, Norway; Faculty of Chemistry, Biotechnology and Food Science, Norwegian University of Life Sciences, Ås, 1430, Norway; Felleskjøpet Fôrutvikling, Trondheim, 7018, Norway; Department of Production Animal Clinical Sciences, Faculty of Veterinary Medicine, Norwegian University of Life Sciences, Ås, 1430, Norway; Department of Paraclinical Sciences, Faculty of Veterinary Medicine, Norwegian University of Life Sciences, Ås, 1430, Norway

**Keywords:** Feed, Pig, Reproduction, Tolerance, Toxicity, Selenium

## Abstract

In June 2023, an incident at a Norwegian feed mill caused pig feed being over-supplemented with sodium selenite (NaSe), resulting in feed selenium concentrations ranging from 2.47 to 4.70 mg Se/kg feed from sodium selenite (NaSe), levels exceeding the European Union (EU) regulatory upper limit of total 0.5 mg Se/kg feed (standardized at 88% DM). The feed containing excessive selenium was unknowingly sent out to production farms. Selenium is an essential trace mineral with a narrow dose range between deficiency and toxicity. The National Research Council (NRC) reports selenium requirements at concentrations of 0.15 mg Se/kg feed for sows and finishing pigs, and 0.30 mg Se/kg feed for weanlings, but studies suggest these levels may not always meet requirements in modern swine production systems. This case study aimed to evaluate the effects of excessive dietary selenium intake on reproductive performance in breeding sows and piglet weaning weight. Data were collected from 516 sows (155 TN70 and 361 Landrace sows) across two Norwegian farms between 2022 and 2024, yielding 895 litter records. Mixed effects models were fitted to evaluate nonpregnant outcomes, stillbirths, litter size, and piglet weaning weight. Despite excessive dietary selenium concentrations, no clinical signs of selenosis or adverse reproductive effects were observed. Excessive selenium intake had no significant effect on nonpregnant outcomes, stillbirths, litter size or piglet weaning weight (all *P* > 0.05). Parity significantly influenced reproductive parameters: higher parity sows showed reduced conception rates (*P* = 0.008), larger litters (*P* < 0.001), more stillborn piglets (*P* = 0.033), and higher piglet weaning weights (*P* < 0.001). Litter size also significantly affected outcomes, with larger litters producing more stillborn piglets and lower piglet weaning weights (both *P* < 0.001). The absence of selenium-related effects suggests that, although dietary selenium concentrations exceeded NRC and EU upper limits, the intake remained within a physiologically tolerable range for high-yielding sows and their offspring. These findings highlight the complexity of selenium tolerance for high-yielding breeding sows under modern production systems.

## Teaser

A case study evaluating the effects of a feed mill incident causing excessive dietary selenium in breeding sows on sow reproductive parameters and piglet weaning weights. Findings suggest that dietary selenium tolerance in modern high-producing sows lays well above current permitted limits.

## Introduction

Selenium is an essential trace mineral for humans and animals, playing a key role in antioxidant defence, thyroid hormone metabolism, immune function and cell regulation (Nabi et al. 2020). These functions are mediated through selenoproteins such as glutathione peroxidases, thioredoxin reductases and iodothyronine deiodinases (Nabi et al. 2020; Abali et al. 2021). Through these physiological functions, selenium plays a central role in growth, reproduction and disease resistance (Labunskyy et al. 2014).

In Scandinavia, the soil selenium content is concerningly low and largely unavailable for uptake through crops (Eich-Greatorex et al. 2007). Consequently, low plant selenium content is transferred through the feed chain, resulting in reduced selenium status in livestock and animal-derived foods (Pecoraro et al. 2022). To compensate for this, animal feed is routinely supplemented with selenium.

The margins between selenium deficiency and toxicity (selenosis) are narrow, and symptoms of deficiency may often overlap with those of selenosis (Koller and Exon 1986). Chronic selenosis in livestock is associated with prolonged dietary intake of 5 to 20 mg Se/kg feed, whereas acute selenosis may result from a single intake exceeding 20 mg Se/kg feed (Kim and Mahan 2001a). Koller and Exon (1986) similarly report that acute selenosis can occur at dietary levels of 10 to 25 mg Se/kg feed. Symptoms of chronic selenosis in pigs include hair loss, impaired liver function and reproduction, heart degeneration, hoof lesions, anaemia, reduced feed intake and reduced growth rate (Kim and Mahan 2001a, 2003). Symptoms regarding reproduction include reduced fertility, particularly smaller litters, more stillborn or weak neonates with higher mortality rate (Kim and Mahan 2001b, 2003). Acute selenosis in pigs produces symptoms such as diarrhoea, cramps, ataxia and respiratory failure (Koller and Exon 1986; Pecoraro et al. 2022).

The National Research Council (NRC) (2012) describes adequate selenium requirements at 0.15 mg Se/kg feed for sows and finishing pigs, and 0.30 mg Se/kg feed for weanlings. The described requirements have remained unchanged since 1979 for sows, and since 1988 for weanlings and finishing pigs (Kim and Lindemann 2007; National Research Council 2012). Current European Union (EU) regulations limit total selenium content in animal feed to 0.50 mg Se/kg feed (standardized at 88% DM), of which up to 0.20 mg Se/kg feed may be of organic origin, such as selenomethionine (SeMet). These limits were first set in the 1970s, based on requirement levels described by the NRC (European Commission 1970). Sivertsen et al. (2007) observed that the selenium status in Norwegian weaning pigs may still be subpar based on plasma selenium levels, even when feeding standardized feeds, designed to meet requirements according to NRC recommendations. Additionally, previous studies (Falk et al. 2018; 2019; 2020) have shown a higher antioxidant capacity and improved immune- and inflammatory responses at levels of 0.43 mg Se/kg feed from SeMet and 0.60 mg Se/kg feed from sodium selenite (NaSe). Advancements in genetics and higher productivity have called EU regulatory limits into question. There is growing concern that current limits may not meet the selenium requirements of modern livestock production (Sivertsen et al. 2007; Falk et al. 2020; Pecoraro et al. 2022). Genetic progress has enabled higher growth rates and reproductive performances in modern pig production. During the past two decades, the daily weight gain of Norwegian slaughter pigs has increased by 22%, reaching an average of 1116 g/day in 2024 (Animalia 2011, 2024). Higher production intensity elevates oxidative stress, thereby increasing the need for antioxidants such as selenium (Prunier et al. 2010; Maes et al. 2020). Environmental and physiological stressors can further exacerbate selenium deficiency and (National Research Council 2012). When antioxidant needs are not met, animals may struggle to cope with intensive production systems, leading to increased (Prunier et al. 2010; Dalgaard et al. 2018; Maes et al. 2020).

During the summer of 2023, a feed mill incident in Norway resulted in pig feed being over-supplemented with sodium selenite (NaSe), resulting in selenium concentrations ranging from 2.47 to 4.70 mg Se/kg feed, up to 26 times the EU regulatory limit. The error was not detected until after feed had been distributed to pig farms and used. Extensive data were subsequently collected in collaboration with the feed mill, slaughterhouses, and producers during the incident. These include feed analyses, slaughter -and production records and reproduction data.

This retrospective analysis from an unfortunate real-world incident, provides an opportunity to extract meaningful insight on increased dietary selenium supplementation in sows. Our objective was to use the collected data materials to evaluate how varying selenium concentrations in feed prior to insemination, during gestation and during lactation influenced reproductive outcomes in modern breeding sows and piglet weaning weight.

## Materials and method

### Animal care and use statement

This study did not involve any experimental procedures on live animals. All data were obtained from existing farm production records, with consent from the farm owners. Approval from the Norwegian Food Safety Authority (Mattilsynet), as required under the Norwegian Animal Welfare Act and Regulation on the Use of Animals in Research (FOR-2015-06-18–761), was not necessary due to the nature of this study. The farms involved operate under Norwegian animal welfare regulations.

### Animals and feed

**Figure 1:**
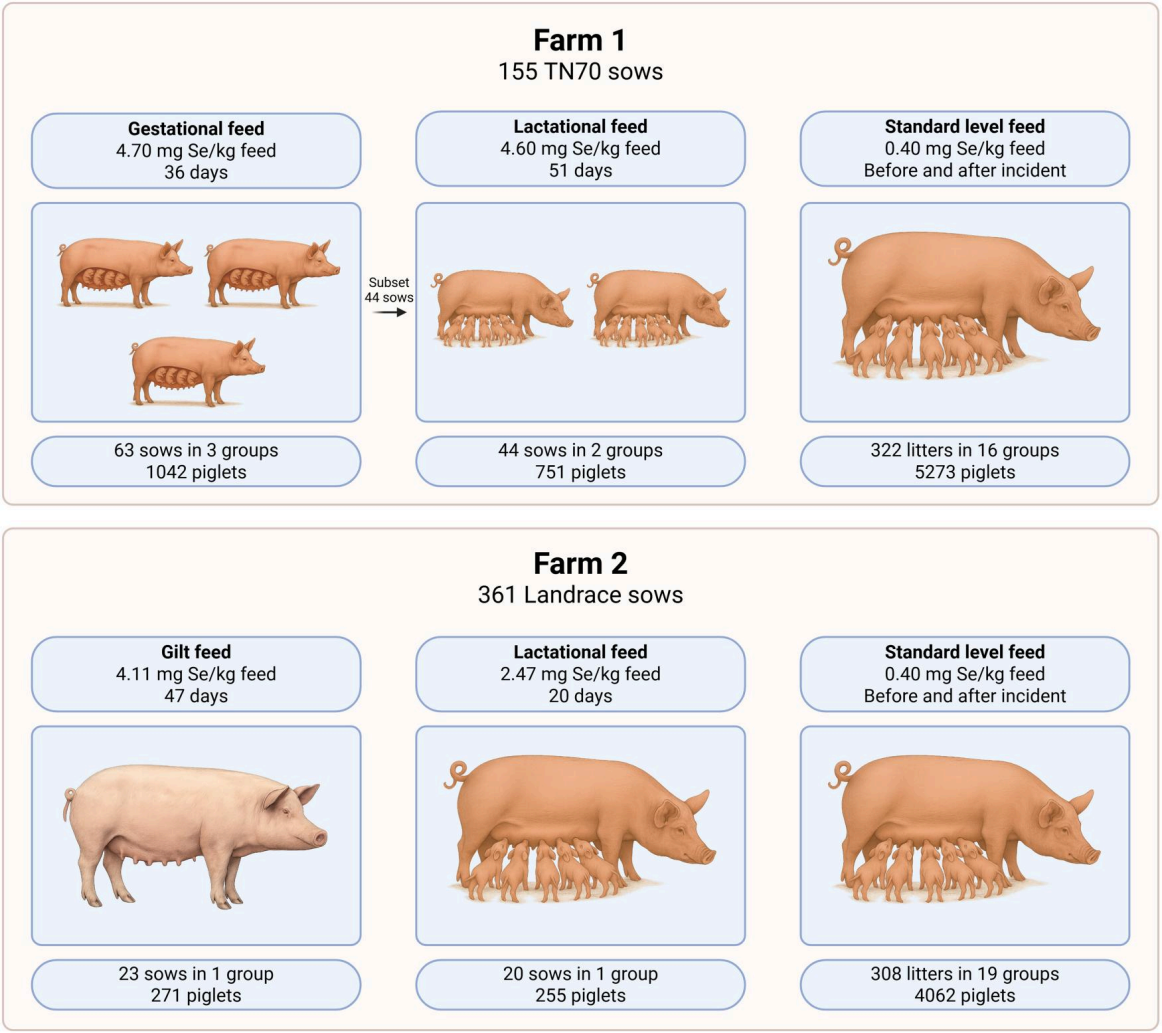
Overview of study design and sample sizes of affected sows on Farm 1 and 2. Summary of the total number of sows (N) and breed from which records were collected, selenium concentrations in gestational, lactational and reference group feed, exposure periods, and sow and piglet counts across groups during the exposure and reference periods. Created in BioRender. Molnes, S. (2026) https://BioRender.com/f4hv7lu

Breeding sows from two farms in Norway (Farm 1 and Farm 2) were included in this analysis. Records were collected from 155 TN70 sows from Farm 1 and 361 Landrace sows from Farm 2 between January 2022 and December 2024. Information on sow age was not available, however parity ranged first to sixth at Farm 1 (mean ± *SD*: 2.80 ± 1.51) and from first to fourth at Farm 2 (mean ± *SD*: 1.26 ± 0.51). During the period from which records were collected, these sows produced multiple litters, resulting in a total of 895 records (429 from Farm 1 and 466 from Farm 2). The study design and distribution of feed exposure periods and sow and piglet counts affected by excessive dietary selenium is summarized in Fig. 1 below.

Cross-fostering between sows is a common practice with larger litters. In our data some sows weaned more piglets than liveborn from the same sow. Unfortunately, information on which piglets originated from which sows and whether piglets were moved to sows of higher or lower parity than their dam were unavailable. However, all sows who have cross-fostered were in gestation and lactation simultaneously, thus receiving approximately equal dietary selenium exposure and accumulation.

The chemical composition of the standard gestational, lactational and gilt feeds, are presented in Table 1.

**Table 1:** Nutrient composition of standard feeds and corresponding affected feeds used at Farm 1 and 2.

	Lactational feed	Gestational feed	Gilt feed
**DM (%)**	87.70	87.00	87.80
**NE, MJ/kg**	9.50	8.90	9.80
**Crude protein, g/kg**	161.00	120.00	182.00
**Crude fat, g/kg**	56.00	52.00	69.00
**Crude ash, g/kg**	55.00	49.00	47.00
**Starch, g/kg**	358.00	346.00	339.00
**Selenium, mg/kg^a^**	0.40 (4.70)	0.40 (4.60/2.47)	0.40 (4.11)
**Phosphorus, g/kg**	8.50	6.40	7.10
**Calcium, g/kg**	4.80	4.10	4.60

a Selenium content of standard feeds from the affected feed mill. Values in parentheses indicate the selenium content in the corresponding error-affected feeds. For gestational feed, content from Farm 1 and Farm 1 are shown separated by a forward slash.

This table shows the nutrient composition of standard feeds produced by the affected feed mill. All nutrients followed the standard formulation, except for selenium. Reference sows at Farm 1 and Farm 1 received these standard feeds. Sows which received erroneously produced feed received the same nutrient composition, except with elevated selenium content at 4.70 mg Se/kg feed for the lactational feed, 2.47 and 4.60 mg Se/kg feed for the gestational feed (Farm 1 and Farm 2 respectively) and 4.11 mg Se/kg feed for the gilt feed (pre-insemination). Inorganic selenium in the form of sodium selenite (NaSe) was used as the selenium source in all feeds.

On Farm 1, pregnant sows received gestational feed containing 4.70 mg Se/kg feed over a period of 36 days. Lactating sows on Farm 1 received lactational feed containing 4.60 mg Se/kg feed for 51 days.

On Farm 2, lactating sows received lactational feed containing 4.11 mg Se/kg feed over a period of 20 days. Gilts on Farm 2 received feed containing 2.47 mg Se/kg feed for a total of 47 days.

Data on daily feed intake (MJ of digestible energy (DE) per day) for Farms 1 and 2 were provided by their respective feed consultants. Sows in early to mid-gestation received 26.5 MJ of DE/day, which increased to 30 MJ of DE/day three weeks prior to parturition. Lactating sows received 66 MJ of DE/day. According to the farmers, sows in both herds consumed the entirety of feed offered, with no observed feed refusal.

Since energy allowance varied across reproductive stages, the amount of selenium each sow consumed also differed. To contextualize this variation, a measure of total dietary selenium exposure, further referred to as DSE, was created. Dietary selenium exposure represents the total amount of selenium consumed by each sow during the exposure period. This metric quantifies the differences in daily feed allowance, Se/kg feed in each feed and number of exposure days, as some sows received excess selenium in only early gestation, others in only late gestation, and others in both gestation and lactation. The calculation of DSE in this analysis is therefore defined as follows:


$$\mathrm{DSE}=\frac{MJ\;of\;DE/\mathrm{day}}{MJ\;of\;DE/kg\;\mathrm{feed}}\;\times \;mg\;\mathrm{Se}/\mathrm{kg}\;\mathrm{fee}d\;\times \;n\;\mathrm{exposure}\;\mathrm{days}$$

As required by Norwegian law, the feed mill collects and stores one representative sample from each produced feed batch, for use in the event of unsuspected production errors. The feed recipient (producer) is also obligated to keep a corresponding feed sample available for analysis if needed. When the fault in the production line was identified, these archived samples were submitted for analysis to Eurofins (Eurofins Food & Feed Testing Norway, Moss, Norway) during the summer of 2023. Sample preparation was performed in accordance with DS/EN 13805:2014, and selenium quantification was carried out using Inductively Coupled Plasma Mass Spectrometry (ICP-MS) according to DS/EN 17294:2016.

### Health and production

Reports from Ingris (Animalia, Norway), a web-based management and reporting tool for Norwegian swine production, were collected for the period of January 2022 to December 2024 for Farm 1 and Farm 2. These reports contain farm identity, sow identity, combined farm-sow identity, parity, insemination dates, date of loss due to abortion, date of return to oestrus in the case of failed conception (non-pregnant), parturition date, number of live born piglets, number of stillborn piglets, weaning date, number of piglets weaned and total litter weaning weight. Records from sows both before and after the incident in June 2023 were collected to serve as control groups. For sows with multiple litters, repeated measures were used with each sow’s earlier and later litters.

Additionally, the producer on Farm 1 submitted mean slaughter production reports from Ingris for three slaughter groups (418 progenies of affected sows) and mean reports for years 2017–2025 (880 progenies of non-affected sows). These reports include key mean numbers for economy, feed, and slaughter results for each slaughter group.

When the feed production error was identified, the animals received immediate veterinary attention. These health inspections were conducted not as part of the current study, but to assess animal health and welfare in relation to the suspected toxicity event. At the time of inspection, the extent of exposure, both in terms of selenium concentration and intake duration, was still unknown. No outward clinical signs consistent with selenosis were observed in animals at Farm 1 and 2 according to the veterinarians who performed the inspections.

### Statistical analysis

All analyses and visualization were performed using *RStudio* (R Core Team 2025). All model assumptions were checked, and P-values were considered significant at 0.05 level.

To assess the effect of excessive selenium exposure prior to insemination on the likelihood of a non-pregnant outcome, a generalized linear mixed model (GLMM) was fitted using the *glmmTB* package in R with the specification ′family = binomial′. The response variable, non-pregnant, was modelled as a binary outcome. The model equation is as follows:


$$\begin{array}{c} l\mathrm{ogit}(p_{i})=\beta_{0}+\beta_{1}\cdot \mathrm{Parit}y_{i}+\beta_{2}\cdot \mathrm{Previous}{\mathrm{DSE}}_{i}+u_{\mathrm{sowID}[i]}\\ \text{Where}:u_{\mathrm{sowID}[i]}∼N(0,\sigma_{\mathrm{sowID}}^{2}) \end{array}$$

The probability (*p_i_*) that a sow had a non-pregnant outcome in observation *i* follows a binomial distribution with a logit link function. The log-odds of a non-pregnant outcome were expressed as a linear function of parity ($\mathrm{Parit}y_{i}$) and DSE prior to insemination ($\mathrm{Previous}\;{\mathrm{DSE}}_{i}$). Sow identity was included as a random intercept ($u_{\mathrm{sowID}[i]}$) to account for repeated measures within sows. Only Farm 2 used feed with excessive selenium prior to insemination, therefore records from Farm 1 were excluded from this model. Non-pregnant sows were excluded, for the remaining analysis.

Further, to evaluate the effect of excessive selenium feed during gestation on litter size a GLMM was fitted using the *glmmTB* package in R with the specification ‘family = gaussian’. The response variable, litter size, was treated as a continuous variable. The model equation is as follows:


$$\begin{array}{c} L\mathrm{itter}\;\mathrm{siz}e_{i}=\beta_{0}+\beta_{1}\cdot \mathrm{Parit}y_{i}+\beta_{2}\cdot {\mathrm{DSE}\;\mathrm{Gestation}}_{i}+u_{\mathrm{sowID}[i]}+\varepsilon_{i}\\ \text{Where}:u_{\mathrm{sowID}[i]}∼N(0,\sigma_{\mathrm{sowID}}^{2}),\varepsilon_{i}∼N(0,\sigma^{2}) \end{array}$$

The litter size in observation *i* was modelled using a gaussian distribution. The expected litter size ($\mathrm{Litter}\;\mathrm{siz}e_{i}$) was expressed as a linear function of parity ($\mathrm{Parit}y_{i}$) and DSE during gestation (${\mathrm{DSE}\;\mathrm{Gestation}}_{i}$). Sow identity was included as a random intercept ($u_{\mathrm{sowID}[i]}$) to account for repeated measures within sows. Only Farm 1 used feed with excessive selenium during gestation therefore, records from Farm 2 were excluded from this model.

For analyses of number of stillborn, a GLMM was fitted using the *glmmTB* package in R with the specification ′family = binomial′. The response was specified as a two-column binomial variable, with the number of stillborn and liveborn piglets provided as the two outcomes. The model equation is as follows:


$$\begin{array}{c} S\mathrm{tillbor}n_{i}∼\mathrm{Binomial}(n_{i},p_{i})\\ \begin{array}{c} n_{i}=\mathrm{Stillbor}n_{i}+\mathrm{Livebor}n_{i}\\ \begin{array}{c} \mathrm{logit}\left(p_{i}\right)=\beta_{0}+\beta_{1}\;\cdot \mathrm{Parit}y_{i}+\beta_{2}\;\cdot \mathrm{Litter}\;\mathrm{siz}e_{i}+\beta_{4}\;{\cdot \mathrm{DSE}\;\mathrm{Gestation}}_{i}+u_{\mathrm{sowID}[i]}\\ \text{Where:}\;u_{\mathrm{sowID}[i]}∼N(0,\;\sigma_{\mathrm{sowID}}^{2}) \end{array} \end{array} \end{array}$$

The number of stillborn piglets in observation *i* follows a binomial distribution with size *n_i_* (total born) and probability *p_i_* of a piglet being stillborn. The log-odds (logit) of a piglet being stillborn was modelled as a linear function of parity ($\mathrm{Parit}y_{i}$), litter size ($\mathrm{Litter}\;\mathrm{siz}e_{i}$) and DSE during gestation (${\mathrm{DSE}\;\mathrm{Gestation}}_{i}$). An interaction term between litter size and parity was tested but found to be non-significant and was therefore excluded from the final model. Sow identity was included as a random intercept ($u_{\mathrm{sowID}[i]}$) to account for repeated measures within sows. Only records from Farm 1 were included in this model as Farm 2 did not receive gestational feed with excessive selenium.

To examine the effects of excessive selenium on piglet weaning weight, a GLMM was fit using the *glmmTB* package in R with the specification ′family = gaussian′. The model equation is shown below:


$$\begin{array}{c} M\mathrm{ean}\;\mathrm{weigh}t_{\mathrm{ijk}}=\beta_{0}+\beta_{1}\cdot \mathrm{Parit}y_{\mathrm{ijk}}+\beta_{2}\cdot \mathrm{Litter}\;\mathrm{siz}e_{\mathrm{ijk}}+\beta_{3}\cdot {\mathrm{DSE}\;\mathrm{Gestation}}_{\mathrm{ijk}}+\beta_{4}\cdot {\mathrm{DSE}\;\mathrm{Lactation}}_{\mathrm{ijk}}+v_{\mathrm{sow}[j(k)]}+\varepsilon_{\mathrm{ijk}}\\ \text{Where: }v_{\mathrm{sow}[j(k)]}∼N(0,\sigma_{\mathrm{sow}}^{2}),\varepsilon_{\mathrm{ijk}}∼N(0,\sigma^{2}) \end{array}$$

The mean weight of weaned piglets in observation *i* was modelled using a gaussian distribution. The expected mean weight ($\mathrm{Mean}\;\mathrm{weigh}t_{\mathrm{ijk}}$) was expressed as a linear function of parity ($\mathrm{Parit}y_{\mathrm{ijk}}$), litter size ($\mathrm{Litter}\;\mathrm{siz}e_{\mathrm{ijk}}$), DSE during gestation (${\mathrm{DSE}\;\mathrm{Gestation}}_{\mathrm{ijk}}$) and DSE during lactation (${\mathrm{DSE}\;\mathrm{Lactation}}_{\mathrm{ijk}}$). The inclusion of ${\mathrm{DSE}\;\mathrm{Gestation}}_{\mathrm{ijk}}$ is intended to account for tissue accumulation and potential in-utero transfer of selenium (Burk et al. 2013). An interaction term between litter size and parity was tested but found to be non-significant and was therefore excluded from the final model. The hierarchical structure of the data and repeated measurements on the same sows were accounted for by including a random intercept for sow identity nested within farm ($v_{\mathrm{sow}[j(k)]}$). The response variable, mean weight, was calculated as the total litter weaning weight divided by the number of weaned piglets. Observations with no registered mean weight were removed.

The health and slaughter production records collected from 418 offspring across three slaughter pig groups originating from Farm 1 were reported as group-level means rather than individual-level observations. After consideration, it was concluded that statistical analysis would have limited power and were therefore not performed on these data. Manual inspection of the records showed no evident or concerning outliers.

## Results

### Descriptive statistics

Descriptive statistics in Table 2 summarize sow reproductive performance and DSE across exposure periods, including mean values and standard deviations for liveborn, stillborn and weaned piglets per litter, piglet weaning weight and DSE.

**Table 2:** Sow reproductive performance, piglet outcomes, and dietary selenium exposure (DSE) among affected and reference sows from Farm 1 and Farm 2.

	Farm 1			Farm 2		
*Group*	Gestation	Lactation	Reference	Lactation	Pre-Insemination	Reference
***Live born piglets, n***	16.5 ± 3.2	17.1 ± 2.8	16.4 ± 3.3	12.8 ± 3.3	11.8 ± 4.0	13.2 ± 3.7
***Stillborn piglets, n***	1.1 ± 1.8	0.8 ± 1.1	1.1 ± 1.5	0.7 ± 0.8	1.4 ± 1.8	1.3 ± 1.6
***Weaned piglets, n***	13.1 ± 2.2	13.3 ± 2.3	13.3 ± 2.1	11.0 ± 2.5	10.7 ± 3.6	11.7 ± 2.8
***Piglet Weaning Weight, kg***	11.2 ± 1.2	11.1 ± 1.1	11.3 ± 1.2	12.5 ± 2.3	11.1 ± 2.1	11.6 ± 2.9
***DSE, mg Se***	363 ± 174	597 ± 203	–	332 ± 26	327 ± 172	–

Data are presented as mean ± standard deviation (*SD*). The table summarizes outcomes of sow performance and dietary selenium exposure (DSE) of groups of sows which received feeds containing excessive selenium in different reproductive periods (gestation and lactation in Farm 1; lactation and pre-insemination in Farm 2), along with reference sows from each farm which consumed standard diets (0.40 mg Se/kg). Reproductive outcomes (live-born piglets, stillborn piglets, and weaned piglets per litter) and piglet weaning weights reflect the performance of the sows withing each group. Dietary selenium exposure (DSE) represents the total selenium (mg) consumed throughout the exposure period, for sows which received feed containing excessive selenium.

### Non-pregnant outcomes

From the generalized linear mixed model, parity was associated with non-pregnant outcomes (*P* = 0.008). With an odds ratio above 1 indicating that sows of higher parity had increased likelihood of abortion or returning to oestrous. However, DSE prior to insemination showed no significant impact on non-pregnant outcomes, Table 3.

**Table 3:** GLMM odds ratio estimates of factors affecting non-pregnant outcomes.

	Odds Ratio	95% CI	*P*-value
***Parity***	4.916	[1.515, 1.596]	0.008*
***DSE Pre-Insemination***	1.003	[0.966, 1.010]	0.323

Results from a generalized linear mixed model with binomial distribution estimating effects of parity and accumulated dietary selenium exposure (DSE) prior to insemination on non-pregnant outcomes. Estimates are presented as odds ratios with 95% confidence intervals (CI) and *P*-values. Significant predictors, *P* < 0.05, are indicated with asterisks.

### Litter size

Parity had a positive effect (*P* < 0.001) on litter size (total number born) indicating higher parity sows had larger litters than lower parity sows, with each additional parity increasing the litter size by 0.765 piglets. Accumulated DSE had no effect on litter size, Table 4.

**Table 4:** GLMM Estimates of factors influencing litter size, *n* total born piglets.

	Beta coefficient	95% CI	*P*-value
***Parity***	0.765	[0.541, 0.989]	< 0.001*
***DSE Gestation^a^***	0.0006	[−0.002, 0.003]	0.592

a Values are rounded to three decimal places. Extremely small values are rounded to the nearest meaningful decimal point for clarity.

Results from a generalized linear mixed model estimating the influence of parity and dietary selenium exposure (DSE) during gestation on litter size. Estimates are presented as beta coefficients with 95% confidence intervals (CI) and P-values. Significant predictors, *P* < 0.05, are indicated with asterisks.

**Figure 2:**
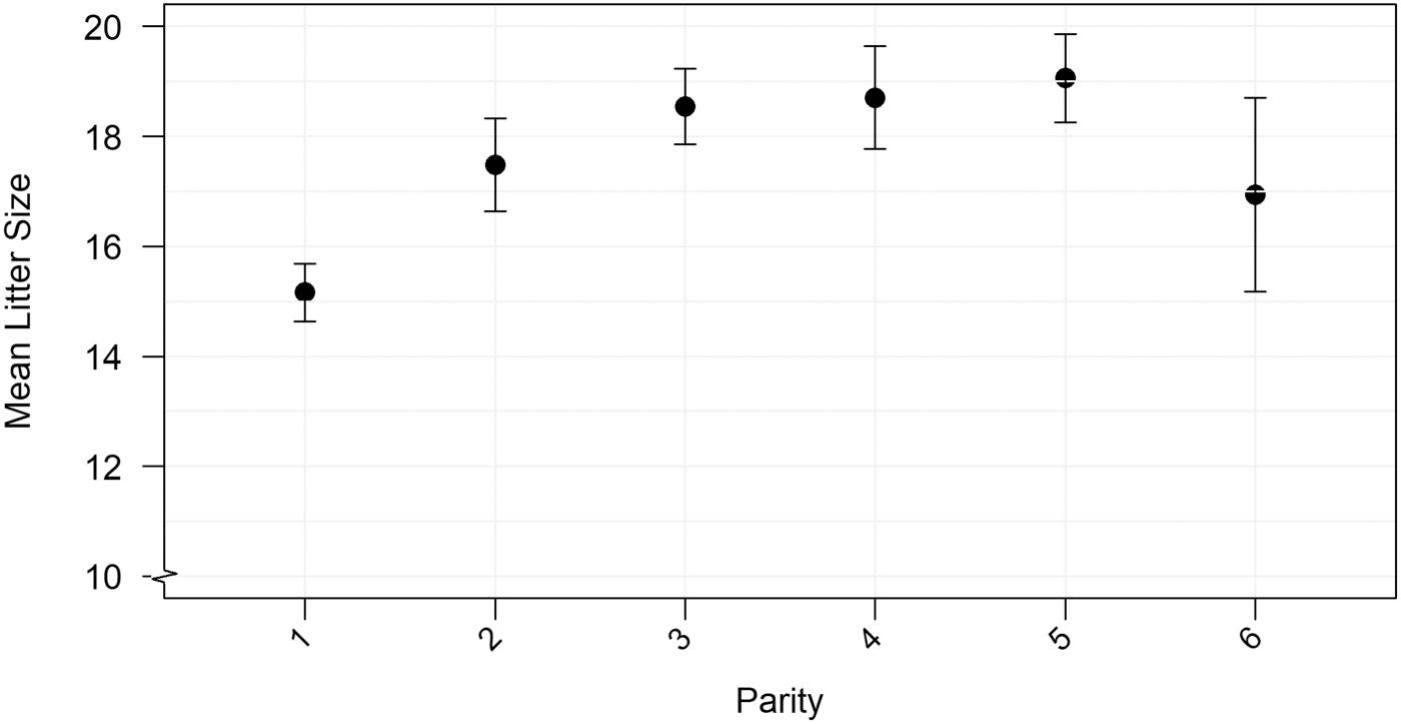
Effect of parity on litter size. The relationship between parity and mean litter size in the data. Each point denotes the observed mean litter size for a given parity, with error bars indicating 95% confidence intervals. The *Y*-axis is truncated at mean litter size of 10 piglets. Pairwise comparisons indicated differences from first parity to second, third, fourth and fifth parity (all *P* < 0.001).

Litter size increased with higher parity (*P* < 0.001), illustrated in Fig. 2. The largest increase seen from first parity to second parity sows, with later litters showing more comparable numbers. Due to the low number of sixth parity sows, the variability and confidence interval is large for this group.

### Stillbirths

The analysis of stillborn piglets revealed statistically significant effects of parity (*P* = 0.033) and litter size (*P* < 0.001). The odds ratio above 1 indicates an increase in incidences of stillbirth with increasing parity and litter size. However, no effect of DSE during gestation was shown on stillborn outcomes, Table 5.

**Table 5:** GLMM Odds ratio estimates of factors affecting stillbirths.

	Odds Ratio	95% CI	*P*-value
***Parity***	1.096	[1.007, 1.192]	0.033*
***Litter size***	1.110	[1.068, 1.153]	< 0.001*
***DSE Gestation^a^***	1.0003	[0.999, 1.000]	0.367

a Values are rounded to three decimal places. Extremely small values are rounded to the nearest meaningful decimal point for clarity.

Results from a generalized linear mixed model with binomial distribution estimating effects of parity, litter size, and dietary selenium exposure (DSE) during gestation on stillbirths. Estimates are presented as odds ratios with 95% confidence intervals (CI) and *P*-values. Significant predictors, *P* < 0.05, are indicated with asterisks.

### Weaning weight

Parity (*P* < 0.001) and litter size (*P* < 0.001) were significant predictors of mean individual weaning weight. For each unit increase in parity weaning weight is estimated to increase by 0.283 kg/piglet, while each additional piglet in the litter is estimated to decrease weaning weight by 0.094 kg/piglet. Neither DSE during gestation nor lactation significantly influenced weaning weight, Table 6.

**Table 6:** GLMM Estimates of factors influencing piglet weaning weight, kg.

	Beta coefficient	95% CI	*P*-value
***Parity***	0.280	[0.158, 0.406]	< 0.001*
***Litter size***	−0.095	[−0.137, −0.053]	< 0.001*
***DSE Gestation^a^***	−0.001	[−0.0026, 0.0006]	0.223
***DSE Lactation^a^***	0.0005	[−0.0006, 0.0017]	0.357

a Values are rounded to three decimal places. Extremely small values are rounded to the nearest meaningful decimal point for clarity.

Results from a generalized linear mixed model estimating the influence of parity, litter size and dietary selenium exposure (DSE) during gestation and lactation on mean piglet weaning weight. Estimates are presented as beta coefficients with 95% confidence intervals (CI) and *P*-values. Significant predictors, *P* < 0.05, are indicated with asterisks.

**Figure 3:**
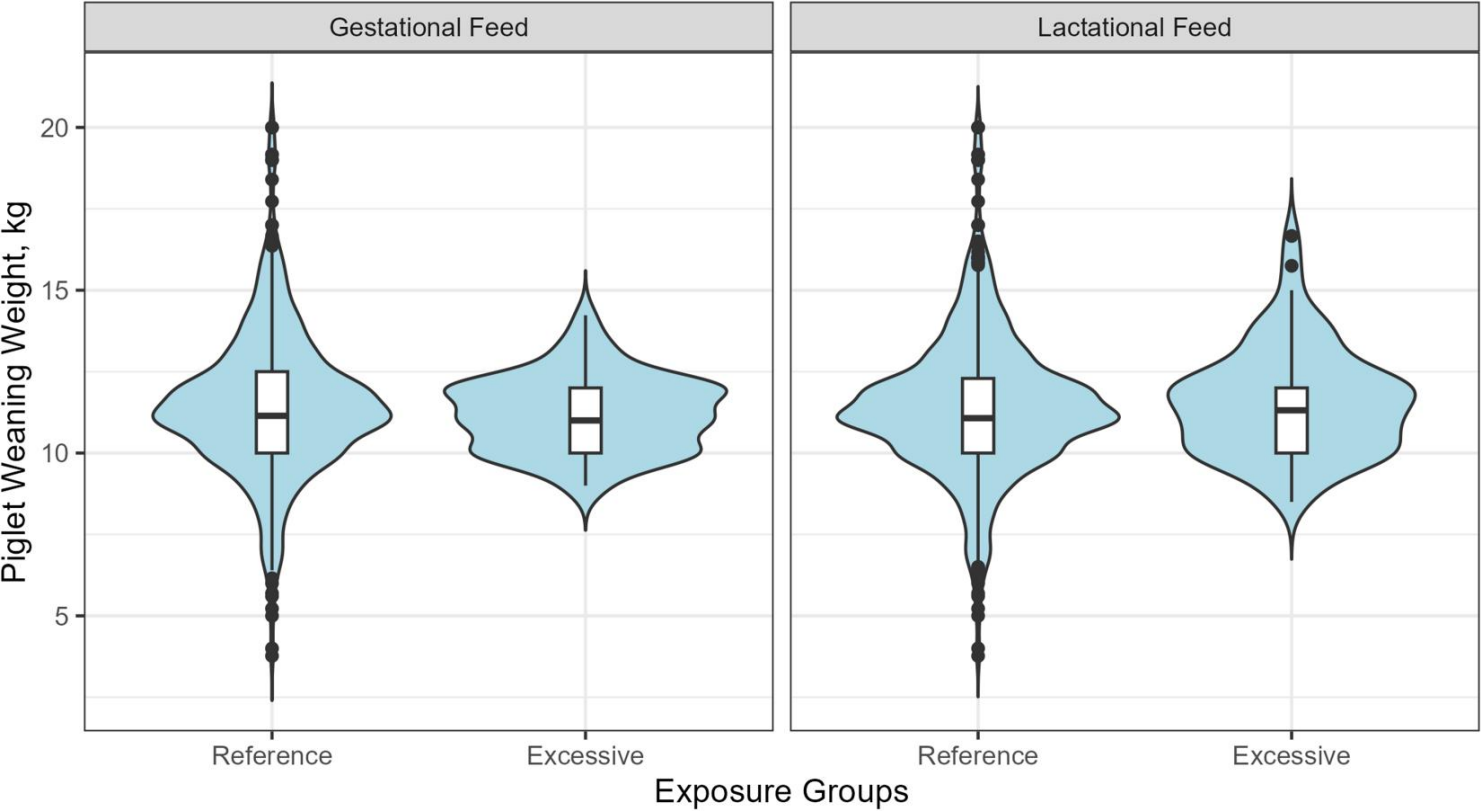
Distribution of piglet weaning weight, kg, during gestation and lactation in sows which received excessive dietary selenium and reference sows. The density of observations on piglet weaning weight, with embedded boxplots indicating the median and variability between reference sows and sows exposed to excessive dietary selenium during gestation or lactation. Pairwise comparisons showed no differences between the groups during gestation nor lactation (*P* > 0.05).

As shown in Fig. 3, the mean piglet weaning weights are comparable between sows fed standard and excessive dietary selenium levels both during gestation and lactation. As shown in Fig. 3, mean piglet weaning weights were similar between sows fed standard and excessive dietary selenium levels during both gestation and lactation. The distributions were broadly comparable across groups; however, the reference groups showed a wider dispersion, with more observations in the tails of the distribution. The higher number of extreme values observed in the reference groups may partly reflect the larger sample size, which increases the likelihood of capturing observations from the tails of the distribution.

## Discussion

The threshold for toxic selenium intake in livestock has been defined at 5 mg Se/kg feed (Koller and Exon 1986), with chronic selenosis typically occurring at 5 to 20 mg Se/kg feed over several weeks to months (Koller and Exon 1986; Kim and Mahan 2001a). In this study, the affected sows received 2.47 to 4.70 mg Se/kg feed from inorganic NaSe. These concentrations were well above the EU upper limits, but remained below those associated with chronic toxicity.

Reproductive outcomes are often discussed in relation to both selenium deficiency and toxicity, with reported effects including reduced conception rates, impaired fertility and increased piglet mortality (Kim and Mahan 2001a; Dalgaard et al. 2018; Falk et al. 2020). Both deficient and excess selenium intake have been associated with adverse pregnancy outcomes, including miscarriage, pre-eclampsia, gestational diabetes, foetal growth restriction, and preterm birth, in both vertebrates and invertebrates (Dahlen et al. 2022). However, human populations exposed to high dietary selenium (up to 1.4 mg Se/day in seleniferous areas) do not consistently exhibit an increased susceptibility to adverse effects during pregnancy (EFSA NDA Panel 2023). In this study, excessive selenium intake showed no measurable effects across reproductive parameters. However, parity significantly influenced non-pregnant outcomes, litter size, and weaning weight, with litter size also affecting weaning weight.

Many adverse effects associated with selenium deficiency stem from an insufficient supply of antioxidants, such as glutathione peroxidase (GPx), which in turn compromises the body’s ability to manage oxidative stress (Labunskyy et al. 2014). On the other hand, excessive selenium can also promote oxidative stress, through catalysation of reactions with thiols leading to generation of reactive oxygen species (ROS) (Stipanuk 2006). Organic selenium can be stored in muscle tissues and released gradually, reducing the immediate selenium availability and subsequent ROS generation (Kim and Mahan 2001b; Stipanuk 2006; Rayman et al. 2008). In contrast, inorganic selenium, is not stored in tissue, resulting in a more rapid metabolism and excretion (Rayman et al. 2008; Falk et al. 2020). Affected sows in this study consumed inorganic selenium at near toxic concentrations over several weeks, however no clinical toxicity or adverse effects on reproductive parameters were observed.

Due to strict regulatory limits and known adverse effects of selenosis, recent studies investigating selenium levels similar to those in this case are rare. Existing literature on levels approaching these is largely limited to older experimental toxicology trials or case reports of accidental over-supplementation, as presented earlier in this article. Moreover, research on selenosis in breeding sows is less available than in finishing pigs.

### Reproductive performance

Parity was an important factor across several reproductive parameters in this study, influencing non-pregnant outcomes, litter size, stillbirths, and weaning weight. These findings are consistent with previous studies showing that sow productivity is highly influenced by parity, with second- and third-parity sows generally weaning more piglets than gilts and older sows (Lavery et al. 2019). Additionally, higher weaning weights in piglets of smaller litters and those suckling higher parity dams are also observed by Bergstrom et al. (2011). The increase in stillbirths and larger litter sizes associated with higher parity sows in our data, reflects patterns described by both Bergstrom et al. (2011) and Lavery et al. (2019). These effects seen in our data, align closely with previous literature, and were thus assumed not to be related to the sows excessive selenium exposure.

### Non-pregnant outcomes

Excessive selenium intake prior to insemination did not affect conception or abortion rates in this study. While literature on the effects of dietary selenium levels above requirement on fertility is limited, decreased conception rates has been observed in swine, cattle and sheep in some experimental and field cases (Wahlstrom and Olson 1959; Raisbeck 2000), while other studies demonstrate no measurable differences even at higher concentrations of inorganic selenium, up to 16 mg Se/kg feed from NaSe (Poulsen et al. 1989). Studies in cattle suggest that selenium supplementation of marginally deficient cattle may not directly impact conception rates, but can reduce offspring mortality (Spears et al. 1986). These findings indicate that while excess selenium has been associated with impaired reproduction in some contexts, the relationship is not consistent across species, dietary level, or experimental conditions.

### Litter size

Excessive selenium intake during gestation had no effect on litter size in this study. This aligns with the broader literature, which reports mixed outcomes depending on selenium concentration, form (inorganic vs. organic), and supplementation method. For example, repeated intramuscular injections of 3 mg Se with 408 IU d-α-tocopherol on days 30, 60 and 100 of gestation resulted in increased litter size and birth weight, despite no changes in other biochemical markers (Chavez and Patton 1986). In contrast, a single injection of NaSe at 0.05 mg Se/kg BW resulted in no observed effects on litter size (Jensen et al. 1984). Dietary experiments also demonstrate differences between selenium forms; sows fed 0.30 mg Se/kg feed from selenium enriched yeast produced larger litters and weaned more piglets than those receiving the same selenium concentration from NaSe (Chen et al. 2016). Similarly, organic selenium provided as HMSeBA at 0.30 mg Se/kg feed has been associated with increased litter size (Mou et al. 2020).

Outcomes with higher dietary selenium concentrations also vary. Inorganic NaSe at 10 mg Se/kg feed has been associated with reduced litter size, whereas concentrations as high as 16 mg Se/kg feed have shown no adverse effects (Wahlstrom and Olson 1959; Poulsen et al. 1989). These discrepancies may reflect differences in baseline selenium status, as animals with marginal deficiency appear to be more responsive to supplementation (Jensen et al. 1984).

Oxidative stress may also be an important mediator, as low antioxidant capacity during gestation and lactation is known to impair reproductive performance (Falk et al. 2019), while excessive selenium may also induce oxidative stress (Stipanuk 2006), potentially explaining reductions in reproductive performance reported at high dietary concentrations.

### Stillbirth

The analysis of stillbirth revealed no effect of excessive maternal dietary selenium intake during gestation in this study. Stillbirth has historically been investigated more extensively in the context of selenium deficiency than in cases of selenium toxicity, or intake above nutritional recommendations (Koller and Exon 1986; Falk et al. 2019). Reduced neonatal mortality has been reported by Chavez and Patton (1986), whereas effect was observed in the study by Jensen et al. (1984). Similarly, even high dietary intake, up to 16 mg Se/kg feed as NaSe, had no effect on neonatal mortality or piglet survival up to nine weeks of age, despite supplementing within an acutely toxic range of selenium (Poulsen et al. 1989). Supplementation with organic selenium (as HMSeBA) at 0.30 mg Se/kg feed reduced both piglet birth interval and total farrowing duration (Mou et al. 2020), factors strongly associated with piglet mortality (Schoos et al. 2023). Together, these findings suggest that selenium’s influence on stillbirth is dependent on many varying factors. Beneficial effects are most apparent when supplementing deficient animals (Jensen et al. 1984), while differences between inorganic and organic selenium, and injections versus dietary supplementation, likely contribute to variable responses across studies.

### Weaning weight

Selenium status and performance of piglets is influenced by the selenium status of the dam (National Research Council 1998). Higher-parity sows, often producing larger litters (Lavery et al. 2019), show reduced selenium concentrations in colostrum and milk, and thereby lower transfer of selenium to their offspring (Pecoraro et al. 2022). Piglets with poor antioxidant status prior to weaning are at increased risk of deficiencies post-weaning (Dalgaard et al. 2018), highlighting the importance of adequate maternal selenium intake for optimal piglet growth and health (Doan et al. 2020). Weanling pigs and young rats suckling selenium-adequate dams demonstrate improved growth and nutrient utilization compared to those suckling selenium deficient dams (Ewan 1976; Glienke and Ewan 1977). Similarly, piglets from sows supplemented NaSe at 0.60 mg Se/kg feed gained more weight during suckling than those from sows supplemented NaSe at 0.40 mg Se/kg feed (Falk et al. 2020). However, maternal dietary selenium intakes of 7 to 10 mg Se/kg feed may be associated with adverse effects on progeny (Kim and Mahan 2001b). In the present study, excessive maternal selenium intake (2.7 to 4.7 mg Se/kg feed from NaSe) resulted in no adverse effects on piglets, even in large litters with high growth rates. Rapid growth increases production of ROS, leading to increased oxidative stress (Alonso-Alvarez et al. 2007), and heightened requirement for antioxidants, such as vitamin E and selenium (Falk et al. 2020). This may explain that the given excessive dietary intake was within a range of tolerance for high yielding sows and their piglets.

Although NaSe is transferred less efficiently to colostrum and milk than organic selenium, transfer is still observed (Poulsen et al. 1989; Kim and Mahan 2001b; Dalgaard et al. 2018; Falk et al. 2019). In ewes, supplementation of 0.20 to 20.00 mg Se/kg feed from NaSe produced a linear increase in milk selenium concentrations (Davis et al. 2006). Because colostrum and milk were not sampled in this study, it remains uncertain whether the absence of effects of selenium on weaning weight reflects limited selenium transfer or a true absence of influence.

### Limitations

This study is based on observational data, a case study, which introduces multiple limitations to consider when interpreting the results. First, the number of sows that received excessive dietary selenium is much smaller than the reference sows. This imbalance results in greater variability and larger confidence intervals for the excessive selenium group. Additionally, because this is not a controlled feeding trial, this study lacks a proper control group. Instead, data were collected from each farm both before and after the incident, allowing earlier and later litters from each sow to serve as a reference for the affected litters. This method accounts for consistent general management and feeding practices, however it does not control for biological, environmental, and health-related variables such as sow physiological differences (e.g., body condition score, underlying health status, reproductive history), disease pressure, seasonal conditions and subtle management that would typically be standardized in controlled experiments.

A further limitation is that the study includes data from only two farms, which differed in both production stage at exposure and location (though in close proximity and located in the same county). This restricts the generalizability of the findings at herd level, as farm-specific factors such as management, environment, location, which may confound the observed effects. Moreover, some analyses are effectively based on a single farm, further limiting external validity. However, the inclusion of control observations within the same farms allows for within-farm comparisons, which reduces between-farm confounding and strengthens the internal validity of the study. In addition, it should be noted that the study was not prospectively designed but is based on data collected following a feed mill incident. Consequently, the study is subject to the constraints of such data, including limited control over farm-level variation and exposure conditions. These factors should be considered when interpreting the results.

Finally, the selenium level in the recruitment feed was much lower (2.47 mg Se/kg feed from NaSe) than the gestational and lactational feeds (4.7 to 4.5 mg Se/kg feed from NaSe). As a result, analysed effects of non-pregnant outcomes cannot be directly compared to the effects from analyses of stillbirths, litter size and weaning weight, which were influenced by higher selenium levels.

## Conclusion

This retrospective case study found no adverse effects of excessive dietary selenium intake (NaSe) at analysed concentrations of 2.47 to 4.70 mg Se/kg feed in short- to medium exposure (20 to 53 days) on sow reproductive outcomes or piglets weaning weight. Our findings may indicate that high yielding sows, with large litters in modern production systems, could have higher selenium tolerance than historical research have found.

## List of Abbreviations

BW: Bodyweight
DSE: Dietary selenium exposure
DE: Digestible energy
EU: European Union
GLMM: Generalized linear mixed model
GPx: Glutathione peroxidase
NaSe: Sodium selenite
NRC: National Research Council
ROS: Reactive oxygen species
SeMet: Selenomethionine

## References

[txag048-B1] Abali E. E. , Cline S. D., Franklin D. S., Viselli S., Harvey R. A. 2021. Biochemistry. 8th ed. Lippincott Williams & Wilkins, Philadelphia.

[txag048-B13] EFSA NDA Panel (EFSA Panel on Nutrition, Novel Foods and Food Allergens) et al 2023. Scientific opinion on the tolerable upper intake level for selenium. EFSA J. 2023;21:7704. 10.2903/j.efsa.2023.7704 PMC9854220 36698500

[txag048-B2] Alonso-Alvarez C. , Bertrand S., Faivre B., Sorci G. 2007. Increased susceptibility to oxidative damage as a cost of accelerated somatic growth in zebra finches. Funct. Ecol. 21:873–879. 10.1111/j.1365-2435.2007.01300.x

[txag048-B3] Animalia. 2012. Ingris årstatistikk 2011. https://www.animalia.no/contentassets/28e0db72674d496186f0570a9e606fca/arsstatistikk-2011.pdf (Accessed September 18 2025).

[txag048-B4] Animalia. 2024. Ingris Årstatistikk 2024. https://www.animalia.no/globalassets/ingris-arsstatistikk-2024.pdf (Accessed September 18, 2025).

[txag048-B5] Bergstrom J. R. et al 2011. The association of sow and litter characteristics with piglet birth weight and the implications for growth, survival, and carcass characteristics of pigs on a commercial farm. Kansas State University, Kansas.

[txag048-B6] Burk R. F. et al 2013. Maternal-fetal transfer of selenium in the mouse. FASEB J. 27:3249–3256. 10.1096/fj.13-231852 23651543 PMC3714584

[txag048-B7] Chavez E. R. , Patton K. L. 1986. Response to injectable selenium and vitamin e on reproductive performance of sows receiving a standard commercial diet. Can. J. Anim. Sci. 66:1065–1074. 10.4141/cjas86-117

[txag048-B8] Chen J et al 2016. Selenium and vitamin E in sow diets: I. Effect on antioxidant status and reproductive performance in multiparous sows. Anim. Feed Sci. Technol. 221:111–123. 10.1016/j.anifeedsci.2016.08.022

[txag048-B9] Dahlen C. R. , Reynolds L. P., Caton J. S. 2022. Selenium supplementation and pregnancy outcomes. Front. Nutr. 9:1011850. 10.3389/fnut.2022.1011850 36386927 PMC9659920

[txag048-B10] Dalgaard T. S. , Briens M., Engberg R. M., Lauridsen C. 2018. The influence of selenium and selenoproteins on immune responses of poultry and pigs. Anim. Feed Sci. Technol. 238:73–83. 10.1016/j.anifeedsci.2018.01.020 32336871 PMC7173062

[txag048-B11] Davis P et al 2006. Effects of selenium levels in ewe diets on selenium in milk and the plasma and tissue selenium concentrations of lambs. Small Ruminant Res. 65:14–23. 10.1016/j.smallrumres.2005.06.016

[txag048-B12] Doan N et al 2020. Organic selenium supplement partially alleviated diquat-induced oxidative insults and hepatic metabolic stress in nursery pigs. Br. J. Nutr. 124:23–33. 10.1017/S0007114520000689 32116206 PMC7512145

[txag048-B14] Eich-Greatorex S. , Sogn T. A., Øgaard A. F., Aasen I. 2007. Plant availability of inorganic and organic selenium fertiliser as influenced by soil organic matter content and pH. Nutr. Cycl. Agroecosyst. 79:221–231. 10.1007/s10705-007-9109-3

[txag048-B15] European Commission. 1970. Council directive 70/524/EEC of 23 november 1970 concerning additives in feeding‐stuffs. Off. J. Eur. Commun. L. 270:1–17.

[txag048-B16] Ewan R. C. 1976. Effect of selenium on rat growth, growth hormone and diet utilization. J. Nutr. 106:702–709. 10.1093/jn/106.5.702 1262978

[txag048-B17] Falk M et al 2018. Effects of dietary sodium selenite and organic selenium sources on immune and inflammatory responses and selenium deposition in growing pigs. J. Trace Elem. Med. Biol. 50:527–536. 10.1016/j.jtemb.2018.03.003 29673733

[txag048-B18] Falk M et al 2020. Beneficial antioxidant status of piglets from sows fed selenomethionine compared with piglets from sows fed sodium selenite. J. Trace Elem. Med. Biol. 58:126439. 10.1016/j.jtemb.2019.126439 31830704

[txag048-B19] Falk M et al 2019. Effects of sodium selenite and L-selenomethionine on feed intake, clinically relevant blood parameters and selenium species in plasma, colostrum and milk from high-yielding sows. J. Trace Elem. Med. Biol. 52:176–185. 10.1016/j.jtemb.2018.12.009 30732879

[txag048-B20] Glienke L. R. , Ewan R. C. 1977. Selenium deficiency in the young pig. J. Anim. Sci. 45:1334–1340. 10.2527/jas1977.4561334x 606706

[txag048-B21] Jensen A. M. , Jensen P. T., Vinther K., Jessen P., Skov-Jensen E. W. 1984. Effect on perinatal mortality of a single selenium injection to sows and gilts. Acta Vet. Scand. 25:436–444. 10.1186/BF03547258 6395688 PMC8287444

[txag048-B22] Kim B. G. , Lindemann M. D. 2007. An overview of mineral and vitamin requirements of swine in the national research council (1944 to 1998) publications. Prof. Anim. Sci. 23:584–596. 10.15232/S1080-7446(15)31028-7

[txag048-B23] Kim Y. Y. , Mahan D. C. 2001a. Comparative effects of high dietary levels of organic and inorganic selenium on selenium toxicity of growing-finishing pigs. J. Anim. Sci. 79:942–948. 10.2527/2001.794942x 11325201

[txag048-B24] Kim Y. Y. , Mahan D. C. 2001b. Prolonged feeding of high dietary levels of organic and inorganic selenium to gilts from 25 kg body weight through one parity. J. Anim. Sci. 79:956–966. 10.2527/2001.794956x 11325203

[txag048-B25] Kim Y. Y. , Mahan D. C. 2003. Biological aspects of selenium in farm animals. Asian Australas. J. Anim. Sci. 16:435–444.

[txag048-B26] Koller L , Exon J. 1986. The two faces of selenium-deficiency and toxicity—are similar in animals and man. Can. J. Vet. Res. 50:297–306. 3527390 PMC1255217

[txag048-B27] Labunskyy V. M. , Hatfield D. L., Gladyshev V. N. 2014. Selenoproteins: molecular pathways and physiological roles. Physiol. Rev. 94:739–777. 10.1152/physrev.00039.2013 24987004 PMC4101630

[txag048-B28] Lavery A et al 2019. An association analysis of sow parity, live-weight and back-fat depth as indicators of sow productivity. Animal. 13:622–630. 10.1017/s1751731118001799 30017016 PMC6378545

[txag048-B29] Maes D. G. D. , Dewulf J., Piñeiro C., Edwards S., Kyriazakis I. 2020. A critical reflection on intensive pork production with an emphasis on animal health and welfare. J. Anim. Sci. 98:S15–S26. 10.1093/jas/skz362 31784754 PMC7433918

[txag048-B30] Mou D et al 2020. Effect of maternal organic selenium supplementation during pregnancy on sow reproductive performance and long-term effect on their progeny. J. Anim. Sci. 98:1–13. 10.1093/jas/skaa366 PMC7739887 33201223

[txag048-B31] Nabi F et al 2020. Nutraceutical role of selenium nanoparticles in poultry nutrition: a review. Worlds. Poult. Sci. J. 76:459–471. 10.1080/00439339.2020.1789535

[txag048-B32] National Research Council. 1998. Nutrient requirements of swine. 10th rev. ed. The National Academies Press, Washington, DC.

[txag048-B33] National Research Council. 2012. Nutrient requirements of swine. 11th rev. ed. The National Academies Press, Washington, DC.

[txag048-B34] Pecoraro B. M. et al 2022. The health benefits of selenium in food animals: a review. J. Anim. Sci. Biotechnol. 13:58. 10.1186/s40104-022-00706-2 35550013 PMC9101896

[txag048-B35] Poulsen H. D. , Danielsen V., Nielsen T. K., Wolstrup C. 1989. Excessive dietary selenium to primiparous sows and their offspring. I. Influence on reproduction and growth. Acta Vet. Scand. 30:371–378. 10.1186/bf03548012 2640772 PMC8142216

[txag048-B36] Prunier A. , Heinonen M., Quesnel H. 2010. High physiological demands in intensively raised pigs: impact on health and welfare. Animal. 4:886–898. 10.1017/S175173111000008X 22444261

[txag048-B37] R Core Team. 2025. R: A language and environment for statistical computing.R Core Team, Vienna, Austria.

[txag048-B38] Raisbeck M. F. 2000. Selenosis. Vet. Clin. North Am. Food Anim. Pract. 16:465–480. 10.1016/S0749-0720(15)30081-5 11084987

[txag048-B39] Rayman M. P. , Infante H. G., Sargent M. 2008. Food-chain selenium and human health: spotlight on speciation. Br. J. Nutr. 100:238–253. 10.1017/S0007114508922522 18346307

[txag048-B40] Schoos A et al 2023. Relationship between piglets’ survivability and farrowing kinetics in hyper-prolific sows. Porcine Health Manag. 9:37. 10.1186/s40813-023-00332-y 37641115 PMC10464185

[txag048-B41] Sivertsen T. , Vie E., Bernhoft A., Baustad B. 2007. Vitamin E and selenium plasma concentrations in weanling pigs under field conditions in norwegian pig herds. Acta Vet. Scand. 49:1. 10.1186/1751-0147-49-1 17201915 PMC1779789

[txag048-B42] Spears J. W. , Harvey R. W., Segerson E. C. 1986. Effects of marginal selenium deficiency and winter protein supplementation on growth, reproduction and selenium status of beef cattle. J. Anim. Sci. 63:586–594. 10.2527/jas1986.632586x 3759693

[txag048-B43] Stipanuk M. H. 2006. Biochemical, physiological & molecular aspects of human nutrition. In: Stipanuk M. H., editor, Biochemical, physiological & molecular aspects of human nutrition. Saunders Elsevier, St. Louis, MO. p. 1101–1112.

[txag048-B44] Wahlstrom R , Olson O. E. 1959. The effect of selenium on reproduction in swine. J. Anim. Sci. 18:141–145.

